# Link Community Detection Using Generative Model and Nonnegative Matrix Factorization

**DOI:** 10.1371/journal.pone.0086899

**Published:** 2014-01-28

**Authors:** Dongxiao He, Di Jin, Carlos Baquero, Dayou Liu

**Affiliations:** 1 College of Computer Science and Technology, Jilin University, Changchun, China; 2 Key Laboratory of Symbolic Computation and Knowledge Engineering of Ministry of Education, Jilin University, Changchun, China; 3 School of Computer Science and Technology, Tianjin University, Tianjin, China; 4 School of Design, Engineering, and Computing, Bournemouth University, Poole, Dorset, United Kingdom; 5 HASLab, INESC TEC and University of Minho, Braga, Portugal; Universiteit Gent, Belgium

## Abstract

Discovery of communities in complex networks is a fundamental data analysis problem with applications in various domains. While most of the existing approaches have focused on discovering communities of nodes, recent studies have shown the advantages and uses of link community discovery in networks. Generative models provide a promising class of techniques for the identification of modular structures in networks, but most generative models mainly focus on the detection of node communities rather than link communities. In this work, we propose a generative model, which is based on the importance of each node when forming links in each community, to describe the structure of link communities. We proceed to fit the model parameters by taking it as an optimization problem, and solve it using nonnegative matrix factorization. Thereafter, in order to automatically determine the number of communities, we extend the above method by introducing a strategy of iterative bipartition. This extended method not only finds the number of communities all by itself, but also obtains high efficiency, and thus it is more suitable to deal with large and unexplored real networks. We test this approach on both synthetic benchmarks and real-world networks including an application on a large biological network, and compare it with two highly related methods. Results demonstrate the superior performance of our approach over competing methods for the detection of link communities.

## Introduction

Many complex systems in the real world exist in the form of networks, such as social networks, biological networks, Web networks, etc., which are collectively referred to as complex networks. One of the main problems in the study of complex networks is the detection of community structure [1], a subject that keeps attracting a great deal of interest. Although no common definition has been agreed upon, a community within a network is usually defined as a group of nodes that are densely connected with respect to the rest of the network. In the past few years, many different approaches have been proposed to uncover community structure in networks. For review, the interested readers can refer to Ref. [2], [3].

Although previous research towards community detection mainly focused on the community of nodes, several recent works begin to switch the attention to community of links [4]–[10]. The motivation is that link communities are more intuitive than node communities in many real-world networks. This is due to the link usually having a unique identity, while the node tends to have multiple roles. In a social network, for instance, most individuals belong to multiple communities such as families, friends, and co-workers, while the link between a pair of individuals often exists for a dominant reason which may represent family ties, friendship, or professional relationships, et al. Furthermore, the links connected to a single node may belong to several different link communities, thus the node can be assigned to multiple communities of links. Accordingly, overlapping communities of nodes, which is another attractive topic in community detection [11], could be detected as a natural byproduct of link communities.

Recently, a number of approaches to the detection of link communities in graphs have been proposed. For instance, Ahn et al. [4] used hierarchical clustering with a similarity metric between links to build a dendrogram of link communities, which provides a rich hierarchy of structures. Further, in order to obtain the most relevant communities, they introduced a link density function to determine the best level at which to cut the tree. Evans et al. [5], [6] transformed the targeted network into the corresponding line graph based on several types of random walks, and then they detected link communities by applying the existing algorithms for node partitioning on this generated line graph. Kim et al. [7] extended the map equation method [8] originally developed for node communities, by assigning the first level code to each link community while still assigning the second level codes to the nodes, so as to find link communities in networks. Pan et al. [9] detected link communities by a local-based method, which finds each natural community through expanding a selected seed by optimizing a proposed local function. He et al. [10] presented a stochastic process based on a link-node-link random walk to unfold the community structure of links, and then used the local mixing properties of the Markov chain to extract the emerged link communities.

Moreover, in face of the good performance and sound theoretical principles, generative models form a promising class of techniques for identifying communities from networks. Techniques that are being actively researched and developed [12]. Recently, several model-based methods have been proposed, which are based on a blockmodel or its variations, and employ different types of inference algorithms, such as expectation-maximization, nonnegative matrix factorization, and others. However, most of them are focused on the detection of node communities [13]–[19]. We are aware of only one exception, which is the algorithm designed by Ball et al. [20] that considers the detection of link communities. In Ball’s method, the model is parameterized by a set of parameters *θ_iz_*’s, where *θ_iz_* denotes the propensity of node *i* to have links in the *z*-th community. Then, they take *θ_iz_θ_jz_* as the expected number of links in the *z*-th community connecting nodes *i* and *j*. Finally, they fit the parameters of this model using a method of maximum likelihood evaluation based on an expectation-maximization algorithm.

In this work, based on Ball’s model, we propose a new generative model to describe the community structure of links. This model is parameterized by two sets of parameters *ω_z_*’s and *φ_iz_*’s, where *ω_z_* denotes the size of a link community *z*, and *φ_iz_* describes the degree of importance when node *i* forms links in this community. Then, the expected number of links in the *z*-th community connecting nodes *i* and *j* is denoted by *ω_z_φ_iz_φ_jz_*. Compared with Ball’s model, here we introduce an additional set of parameters *ω_z_*’s to characterize the sizes of different communities, aiming to make it more flexible when describing link communities. Thereafter, in order to fit the parameters of this model, we define it as an optimization problem based on squared loss, and solve it by using a technique of nonnegative matrix factorization (NMF). At last, we extend the above method by introducing a strategy of iterative bipartition, namely NMFIB, which can not only determine the number of communities all by itself but also get these results with a high efficiency. Therefore, this combined approach is better suited for discovery in unexplored and large networks. Also of note is that, this iterative bipartition process can be used to improve other model-based methods, such as Ball’s method.

## Methods

In this section, we first introduce a model for the description of link communities in networks, and then present a method based on nonnegative matrix factorization to fit the model parameters. Thereafter, we offer an example to illustrate the method. At last, we extend the above method to a new one that automatically determines the number of communities.

### Generative Model

We define the generative model of link communities, which produces networks with a given number *n* of vertices and *m* undirected edges. Assume that the links can be partitioned into *c* communities using a *soft* community membership variable *R*, where *R_ij_^z^* denotes the probability that link (*i*, *j*) belongs to community *z*, subject to 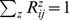. Then the model is parameterized by two sets of parameters *ω_z_*’s and *φ_iz_*’s, where *ω_z_* denotes the size of community z and is defined as twice the expected number of links (or weight) in this community, and *φ_iz_* denotes the probability that community *z* selects node *i* when it generates edges. Thus, we have 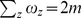 and 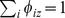.

Based on the above model, an edge (*i*, *j*) can be generated as follows. First, we choose a community *z* randomly with an expected size *ω_z_*. Then by using probabilities *φ_iz_*, *φ_jz_*, community *z* selects nodes *i*, *j* as a pair. Consequently, the expected number of links in community *z,* that lies between nodes *i* and *j*, can be evaluated as
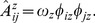
(1)


Summing over communities *z*, the expected number of links between *i* and *j* can be written as
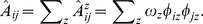
(2)


Note that multiple links and self-edges are both allowed here, which is typical for simple random graph models [18], [20].

Under this model, the link communities will naturally form with the generation of networks. Intuitively, two nodes *i* and *j* which have large values of *φ_iz_* and *φ_jz_*, for some given community *z* with a large size *ω_z_*, should have a high probability of being connected by a link with index *z*. Thus, groups of such nodes will tend to be connected by relatively dense webs of *z*-links, and these sets of edges correctly form the link communities we expect to see.

Formally speaking, assume that the community assignments are represented by a set of variables *R_ij_^z^*’s, where *R_ij_^z^* denotes the fraction by which a link (*i*, *j*) belongs to community *z*. Then we have
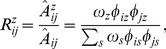
(3)


As the soft membership of communities cannot be used directly, we can simply assign each link (*i*, *j*) to community *r* satisfying *r* = *arg*max*_z_* {*R_ij_^z^*, *z* = 1,2,…,*c*}, and then get the hard partition of links.

### Parameter Fitting

The above model is specified by two sets of parameters *ω_z_*’s and *φ_iz_*’s, depicting, respectively, the constraints 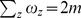 and 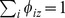. These parameters have to be fitted from the data of the given network *G* to be analyzed. The problem of fitting the model to the data of *G* can be cast as the following optimization problem,



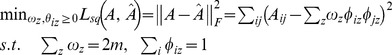
(4)where *L_sq_* is a squared loss function. The best fit between the expected graph with adjacency matrix 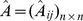 and the given network *G* with adjacency matrix *A* = (*A_ij_*)*_n_*_×*n*_ can be achieved by optimizing (4). In the rest of this section, a method based on nonnegative matrix factorization (NMF) is developed to solve the optimization problem in (4).

We first introduce an auxiliary matrix *X*, where *X_iz_* is defined as



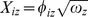
(5)


The loss function in (4) can be rewritten as a constrained nonnegative matrix factorization problem,



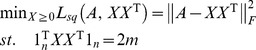
(6)where ||.||*_F_* denotes the Frobenius norm, and 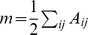. When we get the optimal *X*, using (5) *ω_z_* can be given as
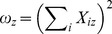
(7)since we have 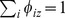, then φiz can be given as



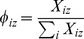
(8)


It is nontrivial to directly optimize (6) with the hard constraints. We relax this optimization problem by introducing a penalty term that represents the hard constraints into the objective function, arriving at minimizing the following objective function,
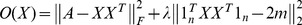
(9)where *λ* is a hyper-parameter that reflects the importance of the hard constraints. Violation to stronger hard constraints incurs a higher penalty to the objective function. In our experiments, we first get an initial value of *X*_0_ by setting *λ*  = 0. Then we restart the optimization with *X* = *X*_0_ and let *λ* to be a relatively large number, e.g. 1000, to minimize the chance of violating the parameter constraints. The purpose of the initialization is to restrict the model search so as to start from some good approximation. Similar to other forms of NMF, the objective function in (9) is not convex w.r.t. *X*, so that it is computationally intractable to find a global minimum. Therefore, a heuristic, gradient descent strategy is adopted to search for local minima. This gradient descent strategy can be implemented in a multiplicative updating algorithm similar to the method for SNMF [13]. In order to derive the update rule, a Lagrange multiplier matrix Θ for the nonnegative constraints on *X* is introduced to (9), resulting in the following equivalent objective function,



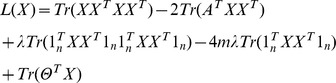



For any stationary state, we have
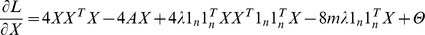



Using complementary slackness condition (Θ)*_iz_*(*X*)*_iz_*  = 0, we have the following equation,
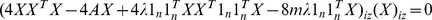



This leads to the following update rule for *X*:
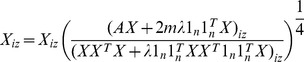
(10)


When the above iteration rule converges, the converged solution satisfies the Karush-Kuhn-Tucker (KKT) conditions [21].

The convergence of the iterative updating rules follows the theorem below.

**Theorem 1:** *The objective function O in (9) is non-increasing under the update rule in (10). O is invariant under these updates if and only if X becomes stationary.*

***Proof:*** We adopt the auxiliary function approach used in Expectation-Maximization and NMF. The basic idea is to construct an auxiliary function 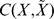 such that:
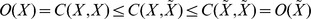



If we can minimize 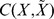 w.r.t to *X*, then we are guaranteed to drive *O*(*X*) down.

Note that,
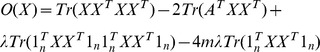





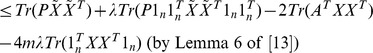


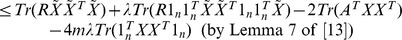


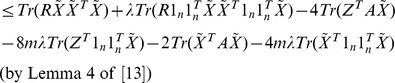





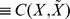
,where 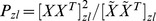, 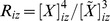, and 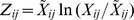. The equality clearly holds when 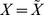. Then 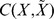 satisfied the conditions of being an auxiliary function for *O*(*X*). We can define the series of update rules as:



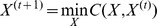


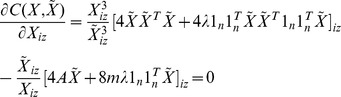



So we get the update rule for *X* as in (10).

Notice that, the time to calculate *AX* in (10) is *mc*, where *m* is the number of edges and *c* is the number of communities. The time to calculate 1*_n_*(1*_n_^T^X*) is *nc*, where *n* is the number of nodes. The time of calculating *X*(*X^T^X*) is *nc* ^2^, and the time of calculating 1*_n_*(1*_n_^T^X*)(*X^T^*1*_n_*(1*_n_^T^X*)) is also *nc* ^2^. Consequently, the time to evaluate (10) once is *O*(*mc+nc* ^2^), and hence the time complexity of our method is *O*(*T*(*mc+nc* ^2^)), where *T* is the iteration number for convergence. Also, according to [20] the time complexity of Ball’s method is *O*(*Tmc*). Therefore, the time complexity of our method is competitive with that of Ball’s since *nc* is often competitive with *m*.

### An Illustrative Example

**Figure 1:**
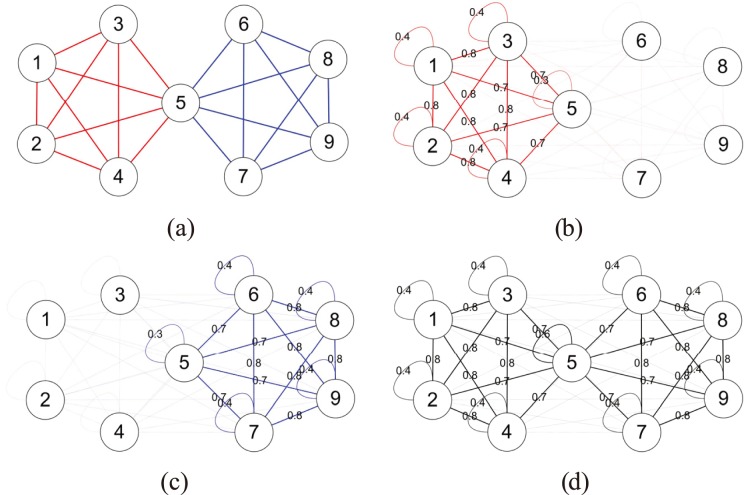
An illustration of our method for identifying the community structure of links. (**a**) The given network *G* with two link communities (in red and blue). (**b**) and (**c**) The expected graph of the red and blue link community. (**d**) The expected graph of *G*, which is an ensemble of the expected graphs of its red and blue communities. Note that the width of a link corresponds to its expected values, and values smaller than 0.1 are omitted.

Here we depict the main idea of our method using a simple example, illustrated by Table 1 and Figure 1.

**Table 1:** The fitted model parameters *ω_z_*’s and *φ_iz_*’s.

		*φ_iz_*								
	*ω_z_*	*i* = 1	*i* = 2	*i* = 3	*i* = 4	*i* = 5	*i* = 6	*i* = 7	*i* = 8	*i* = 9
*z* = 1	19.17899	0.203195	0.203195	0.203195	0.203195	0.175391	0.002957	0.002957	0.002957	0.002957
*z* = 2	20.82149	0.01	0.010855	0.010855	0.010855	0.175391	0.195297	0.195297	0.195297	0.195297

First, we fit the expected graph to the given network *G* by optimizing (4), and get the best parameters of the model *ω_z_*’s and *φ_iz_*’s which are shown as Table 1. Then using *ω_z_*’s and *φ_iz_*’s, we can form the expected graphs of all the link communities in *G* according to (1), which are shown as Figure 1(b) and (c), respectively. Further, we can form the expected graph of the whole network *G* according to (2), which can be regarded as an ensemble of the expected graphs of all its communities, shown as Figure 1(d). Notice that, a value marked between a pair of nodes denotes the expected number (or weight) of links between them. Finally, we can infer the community structure of links according to (3), which is equivalent to dividing the expected graph of the red/blue link community shown in Figure 1(b)/(c) into the expected graph of *G* shown in Figure 1(d). As expected, the result perfectly matches the ground-truth given in Figure 1(a).

### Determining the Number of Communities Automatically

Nonetheless, the method mentioned above can still be improved. The main drawback is that, our model offers no criteria for determining the value of parameter *c*, i.e., the number of communities in a network. This is also a common drawback suffered by almost all methods based on generative models. The statistical model selection applied to generative models may, in principle, be able to find the number of communities in a consistent and satisfactory manner [22], [23], but it is, at present, too computationally demanding to be applied to any but some small networks [20]. Still, it is an open problem whether a reliable method can be developed that runs in reasonable time on the large networks of interest to today’s scientists [18].

Furthermore, even if the number *c* of communities is given, as large networks often have large values of *c*, the convergence rate of the core optimization algorithms (such as expectation-maximization algorithm, nonnegative matrix factorization, et al.) will necessarily become very slow. This is also an important limitation from existing model-based methods when dealing with large-scale networks in the real world.

To mitigate the above problems, we extend our original NMF method proposed above to a more practical one, namely NMFIB, meaning “NMF with iterative bipartition”. In NMFIB, we first divide a network into two link modules using NMF with the number of communities *c*  = 2, and then recursively subdivide the two parts. In dividing a subnetwork, we isolate it from the rest of the network and perform a ‘nested’ NMFIB on it, resulting in a link partition of the subnetwork with two smaller link communities. Subsequently, we decide whether to accept this bipartition by a special method based on the link partition quality. We summarize the algorithm NMFIB using the following recursive algorithm:

**Algorithm** *P* = NMFIB(*G*)//*G* is a network, *P* is a link partition of *G.*

1. *P* = {*E*(*G*)};//*E*(*G*) denotes the edge set of *G.*

2. Divide *G* into two link modules *N*_1_ and *N*_2_ by NMF;

//*E*(*N*_1_)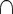 *E*(*N*_2_) = *Φ*, *E*(*N*_1_)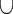 *E*(*N*_2_) = *E*(*N*).

3. If the link partition quality cannot be improved by this bipartition, return *P*;

//the quality function is to be introduced later.

4. *P*_1_ =  NMFIB(*N*_1_);

5. *P*_2_ =  NMFIB(*N*_2_);

6. Return *P* = *P*_1
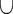
_ *P*_2_.

Subsequently, our remaining task for NMFIB is focused on determining the termination condition for the repetitive process of subdividing the links of network *G*, so as to obtain a superior link community structure. There are several measures for community structures, but most of them are defined for node communities [2], [24], [25]. Fortunately, partition density *D* [4], which is based on a type of link density, is specially designed for link communities. Here we use it as our quality metric, which is introduced as follows.

For a network with *m* links and *n* nodes, *P* = {*P*_1_, *P*_2_, …, *P_c_*} is a partition of the links into *c* communities. The number of links in community *z*, *P_z_*, is *m_z_* = |*P_z_*|. The number of induced nodes, all nodes that those links touch, is *n_z_* = |∪*_eij_*_∈*Pz*,_ {*i*, *j*}|. The link density *D_z_* of *P_z_* is



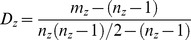
(11)


This is *m_z_* normalized by the minimum and maximum numbers of links among *n_z_* connected nodes. Thus, *D_z_*  = 1 when *P_z_* is a clique, or *D_z_*  = 0 when *P_z_* is a tree. In particular, we assume that *D_z_*  = 0 if *n_z_*  = 2 without loss of generality. In essence, *D_z_* measures how ‘clique-ish’ versus ‘tree-ish’ *P_z_* is. Then, the partition density, *D*, is the average of *D_z_*, weighted by the fraction of links that are present:



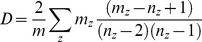
(12)


It is worthy of note that, when using function *D*, the determination of acceptance of the bipartitions for link communities will be independent on the order in which the bipartitioning queue happens in the above iterative procedure. Let we divide an arbitrary community *r* into two sub-communities *r*_1_ and *r*_2_. The community structure will become *P'* = {*P*_1_, …, *P_r_*_-1_, *P_r_*_1_, *P_r_*_2_, *P_r_*_+1_, …, *P_c_*}, and the its *D*-value will be



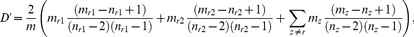
(13)and then the variation of the partition density, denoted by Δ*D*, will be



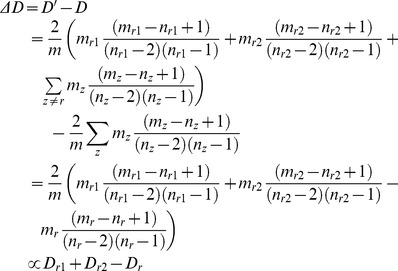
(14)


Then, considering a bipartition for an arbitrary community, the variation of its *D*-value only depends on this community and its bipartition result, and thus the determination of acceptance of the bipartitions for link communities will be independent on the order of the bipartitioning queue.

## Results and Discussion

In order to evaluate the performance of our above method, we tested it both on benchmark synthetic networks and on some widely used real-world networks. The synthetic networks allow us to test the ability of different methods to detect known communities under controlled conditions, while the real networks allow us to observe its performance under practical conditions. Further, we applied our method on a large biological network derived from real world data.

Furthermore, we compared our method with two well-known and highly related methods. The first is a model-based method for link communities proposed by Ball et al [20], and the other is the notable method of link communities proposed by Ahn et al [4]. To the best of our knowledge, our method and Ball’s method are the only two methods based on generative models that handle link communities, and our method and Ahn’s method are the only two hierarchical methods considering partition density *D* [4] as the cut metric to detect link communities.

All experiments are done on a single Dell Server (Intel(R) Xeon(R) CPU 5130 @ 2.00 GHz 2.00 GHz processor with 4 Gbytes of main memory). The source code of the algorithms used here can all be obtained from the authors. Especially, the code of our methods, which are written as two functions NMF (our original method) and NMFIB (our method with iterative bipartition) in Matlab, is available in [26]. Also, interested researchers can contact us directly if interested on the code and instructions.

### Synthetic Networks

Recently, several types of synthetic benchmarks have been proposed for node communities [1], [27], [28]. However, there is only one benchmark, to our knowledge, designed for testing the fitness of algorithms with respect to link community detection [20], and thus it is the one selected for use in this evaluation. Furthermore, we employed two accuracy measures introduced in [20], namely “Fraction of Vertices Classified Correctly (FVCC)” and “Jaccard index”, to compare the planted community structure of the network and the one delivered by the algorithm. Notice that Anh’s method does not appear here, this because it often finds very small communities, and fails to detect the communities defined in this benchmark.

As done in the experiment designed by Ball et al. in [20], the parameter setting for this benchmark is given as follows. The networks have *n*  = 10000 nodes each, divided into two overlapping (link) communities. We placed *x* nodes in the first community only, i.e., these nodes have connections exclusively within the community, *y* nodes in the second community only, and the remaining *z* = *n*-*x*-*y* nodes in both communities, with equal numbers of connections to nodes in these two communities on average. We set the expected degree of all nodes to a fixed value <*k>*. We also varied the parameters *x*, *y*, *z*, and <*k>* to generate networks with stark community structures or no structure at all, so as to vary the difficulty of the network instances posed to the algorithms.

We performed three sets of tests. In the first set of experiments, we fixed the size of the overlap between the communities at *z*  = 500, divided the remaining nodes evenly (i.e., *x* = *y*  = 4750), and varied the value of <*k>* from 1 to 16 with an increment of 1. For the second set of tests, we again set the overlap at *z*  = 500 but fixed <*k>*  = 10 and varied the ratio between *x* and *y*. Finally, for the third set of tests, we set <*k>*  = 10, constrained *x* and *y* to be equal, and varied the amount of overlap *z*.

**Figure 2:**
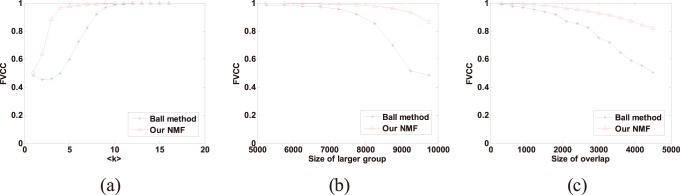
Comparison of our NMF method and Ball’s method in the three sets of synthetic networks, measured by the fraction of vertices classified correctly (FVCC). Each data point in the figure is an average over 50 network instances. (**a**) FVCC accuracy as a function of the expected degree <*k*> of all nodes. (**b**) FVCC accuracy as a function of the size of the larger community. (**c**) FVCC accuracy as a function of the amount of overlap between the two communities.

As the actual number of communities of the benchmark networks used here is known to be 2, for fairness we make it as a *priori* information for both our NMF method and Ball’s method in this comparison. In Figure 2, we show the fraction of correctly classified nodes by the two algorithms for each of the three sets of experiments. To be considered correctly classified, a node’s membership in both communities must be reported correctly by an algorithm. As shown in Figure 2, our NMF method outperforms Ball’s method in terms of FVCC accuracy in all the three tests.

**Figure 3:**
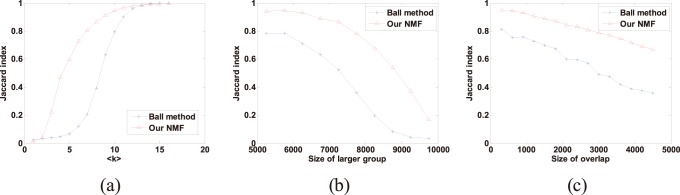
Comparison of our NMF method and Ball’s method in the three sets of synthetic networks measured by the Jaccard index. Each data point in the figure is an average over 50 network instances. (**a**) Jaccard index as a function of the expected degree <*k*>. (**b**) Jaccard index as a function of the size of the larger community. (**c**) Jaccard index as a function of the amount of overlap.

Furthermore, we adopted the Jaccard index to compare the two algorithms’ ability for identifying overlapping (link) communities using the same sets of network instances. Let *S* be the set of truly overlapping nodes and *V* be the set of predicted overlapping nodes, the Jaccard index is *J* = |*S*∩*V*|/|*S*∪*V*|. This index is a standard measure of similarity between sets that rewards accurate identification of the overlap while penalizes both false positives and false negatives. Figure 3 shows the result of comparing the two algorithms using the Jaccard index. As shown, our NMF method is also superior to Ball’s method in all the three sets of experiments. This result is similar to the results in Figure 2, and they both confirm the validity of our method.

### Real Networks

As real-world networks may have some unique topological properties not present in synthetic ones, we now consider some widely used real networks to further evaluate the performance of these algorithms. All the networks we used here are obtained from Newman’s website [29], except that ‘protein-protein interaction’ and ‘word association’ introduced by [11] are got from Palla’s website [30]. Besides, as some of the compared methods partly optimized the partition density *D* [4], it seems to be unreasonable if we adopt *D* as the quality metric to compare their results. Fortunately, the extended map equation [7], which is based on the principle of minimum description length (MDL) [31], can naturally measure link communities. Therefore, we used it to evaluate community structures obtained by different methods. Under this measure, the shorter the MDL of an overall community structure, the better the structure is.

In the following, in order to evaluate these methods fairly and completely, we perform three sets of tests. First, we use the number of communities attained by Ahn’s method as a *priori* information needed by Ball’s method and our NMF method, and compare these three methods under the condition that the number of communities is the same. The compared results are shown in Table 2. As we can see, our NMF method has the best performance on 12 of the 15 networks in terms of MDL, and Ahn’s method performs best on the other 3 networks. Note that our NMF method and Ball’s method both do not get the result on the largest network ‘word association’ within the limited time and memory.

**Table 2:** Comparison of algorithms for detecting link communities on some real networks.

			MDL-values		
Datasets [29], [30]	n	m	Ball’s method	NMF	Ahn’s method
Zachary’s karate club	34	78	5.3048	**4.8688**	5.2200
Dolphin social network	62	160	6.5674	**5.6924**	6.1854
High school friendship	69	220	6.5176	**5.6402**	5.9488
Les Miserables	77	254	5.4211	**5.0129**	5.3196
Political books	105	441	7.8538	**6.9102**	7.1717
Word adjacencies	112	425	8.2971	7.5387	**7.3043**
American college football	115	613	7.8748	**6.9505**	7.1398
Jazz musicians collaborations	198	2,742	8.9908	8.5043	**8.0360**
C. Elegans neural	297	2,148	11.0720	**9.8216**	10.6335
E. coli metabolic	453	2,025	9.0538	**8.5349**	9.7428
E-mail network URV	1,133	5,451	11.7156	**10.2404**	11.7598
Political blogs	1,490	16,717	14.2742	12.7867	**12.1782**
Network science collaborations	1,589	2,742	4.0705	**3.9230**	4.1812
Power grid	4,941	6,594	7.4667	**6.5839**	8.9819
Protein-protein interaction	2,640	6,600	9.8311	**8.5575**	9.8867
Word association	5,017	29,148	−	−	14.5691

Here, Ball’s method and our NMF method both used the number of communities *c* got by Ahn’s method as a *priori* information. In the table, ‘−’ denotes run time >48 hours or triggering of out-of-memory conditions.

Further, we compared the performance of our NMF method with iterative bipartition (NMFIB), Ball’s method with iterative bipartition, as well as Ahn’s method. At this time, all these methods can determine the number of communities automatically. The comparison of these algorithms is shown in Table 3. We find out that, our method NMFIB has the best performance on 13 of the 16 networks in terms of MDL, Ball’s method performs best on one network, and Ahn’s method performs best on the two remaining networks.

**Table 3:** Comparison of algorithms for detecting link communities on some real networks.

	MDL-values		
Datasets	Ball’s method with IB	NMFIB	Ahn’s method
Zachary’s karate club	**5.2072**	5.2117	5.2200
Dolphin social network	5.4079	**5.0291**	6.1854
High school friendship	5.9890	**5.5903**	5.9488
Les Miserables	5.2863	**5.1267**	5.3196
Political books	6.8298	**5.9248**	7.1717
Word adjacencies	7.1179	**6.3625**	7.3043
American college football	7.0854	**6.6730**	7.1398
Jazz musicians collaborations	8.7322	**7.6698**	8.0360
C. Elegans neural	10.0340	**8.4119**	10.6335
E. coli metabolic	8.9949	**8.7959**	9.7428
E-mail network URV	10.4825	**10.0901**	11.7598
Political blogs	10.7068	**9.4971**	12.1782
Network science collaborations	4.2796	4.2834	**4.1812**
Power grid	10.5560	9.8559	**8.9819**
Protein-protein interaction	8.7585	**8.6888**	9.8867
Word association	12.8988	**12.0587**	14.5691

Here, Ball’s method and our NMF method both use the strategy of iterative bipartition (IB) to automatically determine the number of communities.

Finally, we compare our NMF method with iterative bipartition (and also Ball’s method with IB) and our original NMF method (and Ball’s original method) with the given number of communities *c* got by the corresponding iterative bipartition method. The compared results are shown in Table 4. As we can see, the clustering quality of the iterative bipartition method is competitive with that of the original method for both our NMF method and Ball’s method. But notice that, the efficiency of the iterative bipartition method is much higher than that of the original one.

**Table 4:** Comparison of algorithms for detecting link communities on some real networks.

	MDL-values		MDL-values	
Datasets	Ball’s method with IB	Ball’s method	NMFIB	NMF
Zachary’s karate club	5.2072	**4.9334**	5.2117	**4.8270**
Dolphin social network	5.4079	**5.3942**	5.0291	**4.8292**
High school friendship	5.9890	**5.9878**	5.5903	**5.4040**
Les Miserables	5.2863	**5.2747**	5.1267	**5.0930**
Political books	**6.8298**	7.0674	**5.9248**	6.1170
Word adjacencies	7.1179	**7.1070**	**6.3625**	**6.3625**
American college football	**7.0854**	7.2495	**6.6730**	6.7889
Jazz musicians collaborations	**8.7322**	8.8752	**7.6698**	8.2161
C. Elegans neural	**10.0340**	10.2420	**8.4119**	8.8232
E. coli metabolic	8.9949	**8.7843**	8.7959	**8.2545**
E-mail network URV	**10.4825**	10.4990	10.0901	**9.2841**
Political blogs	**10.7068**	12.4969	**9.4971**	10.6238
Network science collaborations	4.2796	**4.0358**	4.2834	**3.9118**
Power grid	10.5560	**8.3363**	9.8559	**7.9509**
Protein-protein interaction	8.7585	**8.4761**	8.6888	**7.5986**
Word association	12.8988	−	12.0587	−

Here, the number of communities *c* used by our original NMF method (and Ball’s original method) is got by our NMF method with iterative bipartition (and Ball’s method with IB). In the table, ‘−’ denotes run time >48 hours or triggering of out-of-memory conditions.

To sum up, our method with iterative bipartition not only has a higher clustering quality compared with other methods, but also it can determine the number of communities automatically. Thus, it may be more suitable for use when detecting link communities on unexplored real networks.

### Application

The large real network we selected for a particular application is the protein-protein interaction (PPI) network of budding yeast *Saccharomyces cerevisiae* [11], [32]. It contains 2,640 nodes (proteins) and 6,600 links (physical interactions between pairs of proteins).

We used the Gene Ontology (GO) terms [33], the most elaborate gene function annotations, as domain metadata for quality assessment. The GO terms include information on functions and cellular locations of a gene and biological pathways that a gene may be involved in. The biological significance of a community of genes (nodes) can be measured by the GO terms enriched in the genes in the community. Enrichment of GO terms can be evaluated by a hyper-geometric test [34], providing a GO term a *p*-value to quantify the significance of the term. To quantify the biological significance of a community structure, we used as quality metric the average number of significantly enriched GO terms with *p*-values less than a given threshold for all communities. The larger this average number of significant GO terms, the more biologically significant the community structure is.

**Figure 4:**
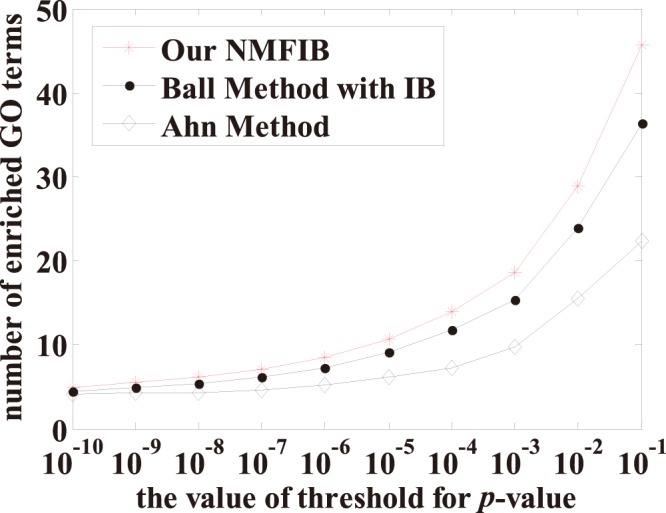
Comparison of our method NMFIB, Ball’s method with iterative bipartition (IB) and Ahn’s method on budding yeast PPI network.

Here we compared the results of our method NMFIB, Ball’s method with iterative bipartition and Ahn’s method, since all of them can automatically determine the number of communities. The results are shown in Figure 4. As we can see, our NMFIB identified PPI community structures with many more significant GO terms than Ball’s method and Ahn’s method under all 10 different *p*-value thresholds tested. It stands as an example of the consistent superior performance of our method over all compared competing methods.

## Conclusions

In this work, we proposed a generative model for link communities based on Ball’s model [20]. Compared with Ball’s model, we included an additional set of parameters, *ω_z_*’s, to characterize the sizes of different communities, which may enable our model to be more flexible in describing link communities. Then, we fitted the model parameters by taking it as an optimization problem, and used an approach of nonnegative matrix factorization to solve it. Thereafter, we extended the above method by introducing a strategy of iterative bipartition. This leads to a new method, namely NMFIB, that we show to be more suitable for structure discovery in unexplored and large networks. Also, this iterative bipartition process is suitable to be used to improve other model-based methods, such as Ball’s method. We tested our method both on synthetic benchmarks and on real-world networks including an application on a large biological network, and compared it with two well-known and highly related methods. Experimental results demonstrated the superior performance of our method over the competing methods in the detection of link communities.

Our model was mainly designed to describe link communities, but it can be also extended to find node communities. A simple and approximate method is assigning each node solely to the community to which it most strongly belongs in the overlapping (link) community structure. But in the future, we wish to improve our model from some other viewpoints, and make it able to describe link communities as well as node communities, naturally and in a similar way, rather than being a simple extension. Furthermore, as discussed, the efficiency of our method is competitive with that of Ball’s method, and it will become more efficient when introducing the strategy of iterative bipartition. Nevertheless, in order to deal with some very large networks such as the WWW, the Internet, etc, its efficiency needs still to be improved. Possibly, we can devise a pruning strategy that sets to zero any *X_iz_* (elements in the auxiliary nonnegative matrix *X*) that falls below a predetermined threshold, and improve the efficiency of our NMF method by using a technique of sparse matrix calculations. This is one of the directions for our future work. Moreover, in the current work, we only used partition density *D* [4] as the metric to determine the acceptance of each bipartition. However, there are some other quality metrics for link communities, such as the extended modularity [5], [6] or the extended map equation [7], which may be also suitable for our iterative bipartition procedure. Thus in the future, we wish to include in our software the option of choosing different quality metrics, which may make our method more powerful.

## References

[1] Girvan M, Newman MEJ (2002) Community structure in social and biological networks. Proc Natl Acad Sci U S A 9: 7821–7826. 10.1073/pnas.122653799 PMC122977 12060727

[2] Fortunato S (2010) Community detection in graphs. Phys Rep 486: 75–174.

[3] Xie J, Kelley S, Szymanski BK (2013) Overlapping community detection in networks: the state of the art and comparative study. ACM Comput Surv 45: article no.43.

[4] Ahn YY, Bagrow JP, Lehmann S (2010) Link communities reveal multiscale complexity in networks. Nature 466: 761–764. 20562860 10.1038/nature09182

[5] Evans TS, Lambiotte R (2009) Line graphs, link partitions, and overlapping communities. Phys Rev E 80: 016105. 10.1103/PhysRevE.80.016105 19658772

[6] Evans TS, Lambiotte R (2010) Line graphs of weighted networks for overlapping communities. Eur Phys J B 77: 265–272.

[7] Kim Y, Jeong H (2011) Map equation for link communities. Phys Rev E 84: 026110. 10.1103/PhysRevE.84.026110 21929067

[8] Rosvall M, Bergstrom CT (2008) Maps of random walks on complex networks reveal community structure. Proc Natl Acad Sci U S A 105: 1118–1123. 18216267 10.1073/pnas.0706851105 PMC2234100

[9] Pan L, Wang C, Xie J, Liu M (2011) Detecting link communities based on local approach. ICTAI’11: Proc. 23rd IEEE Int. Conf. on Tools with Artificial Intelligence (Boca Raton, Florida, USA: IEEE) 884–86.

[10] He D, Liu D, Zhang W, Jin D, Yang B (2012) Discovering link communities in complex networks by exploiting link dynamics. J Stat Mech 2012: P10015.

[11] Palla G, Derenyi I, Farkas I, Vicsek T (2005) Uncovering the overlapping community structures of complex networks in nature and society. Nature 435: 814–818. 15944704 10.1038/nature03607

[12] Newman MEJ (2012) Communities, modules and large-scale structure in networks. Nature Physics 8: 25–31.

[13] Wang F, Li T, Wang X, Zhu S, Ding CHQ (2011) Community discovery using nonnegative matrix factorization, Data Mining and Knowledge Discovery. 22: 493–521.

[14] Psorakis I, Roberts S, Ebden M, Sheldon B (2011) Overlapping community detection using Bayesian non-negative matrix factorization. Phys Rev E 83: 066114. 10.1103/PhysRevE.83.066114 21797448

[15] Zhang Y, Yeung D (2012) Overlapping community detection via bounded nonnegative matrix tri-factorization. KDD’12: Proceedings of the 18th ACM SIGKDD international conference on Knowledge discovery and data mining (Beijing, China: ACM) 606–614.

[16] Ren W, Yan G, Liao X, Xiao L (2009) Simple probabilistic algorithm for detecting community structure. Phys Rev E 79: 036111. 10.1103/PhysRevE.79.036111 19392022

[17] Shen H, Cheng X, Guo J (2011) Exploring the structural regularities in networks. Phys Rev E 84: 056111. 10.1103/PhysRevE.84.056111 22181477

[18] Karrer B, Newman MEJ (2011) Stochastic blockmodels and community structure in networks. Phys Rev E 83: 016107. 10.1103/PhysRevE.83.016107 21405744

[19] Zhang Z, Wang Y, Ahn YY (2013) Overlapping community detection in complex networks using symmetric binary matrix factorization. Phys Rev E 87: 062803. 10.1103/PhysRevE.87.062803 23848725

[20] Ball B, Karrer B, Newman MEJ (2011) Efficient and principled method for detecting communities in networks. Phys Rev E 84: 036103. 10.1103/PhysRevE.84.036103 22060452

[21] Boyd S, Vandenberghe L (2004) Convex optimization. Cambridge, UK: Cambridge University Press.

[22] Brunet J-P, Tamayo P, Golun TR, Mesirov JP (2004) Metagenes and molecular pattern discovery using matrix factorization. Proc Natl Acad Sci U S A 101: 4164–4169. 15016911 10.1073/pnas.0308531101 PMC384712

[23] Tan VYF, Févotte C (2012) Automatic relevance determination in nonnegative matrix factorization with the β-divergence. IEEE Transactions on Pattern Analysis and Machine Intelligence 35: 1592–1605. 10.1109/TPAMI.2012.240 23681989

[24] Newman MEJ, Girvan M (2004) Finding and evaluating community structure in networks. Phys Rev E 69: 026113. 10.1103/PhysRevE.69.026113 14995526

[25] Lancichinetti A, Radicchi F, Ramasco JJ, Fortunato S (2011) Finding statistically significant communities in networks. PLoS ONE 6: e18961. 21559480 10.1371/journal.pone.0018961 PMC3084717

[26] The software of our methods NMF and NMFIB. Available: ftp://jindi:dd@59.72.0.62:2121.

[27] Lancichinetti A, Fortunato S, Radicchi F (2008) Benchmark graphs for testing community detection algorithms. Phys Rev E 78: 046110. 10.1103/PhysRevE.78.046110 18999496

[28] Lancichinetti A, Fortunato S (2009) Benchmarks for testing community detection algorithms on directed and weighted graphs with overlapping communities. Phys Rev E 80: 016118. 10.1103/PhysRevE.80.016118 19658785

[29] Real-world networks we used. Available: http://www-personal.umich.edu/~mejn/netdata/.

[30] Networks ‘protein-protein interaction’ and ‘word association’. Available: http://www.cfinder.org/.

[31] Grünwald PD (2007) The minimum description length principle. Cambridge, Massachusetts, USA: The MIT Press.

[32] Xenarios I, Rice DW, Salwinski L, Baron MK, Marcotte EM, et al (2000) DIP: the database of interacting proteins. Nucleic Acids Research 28: 289–291. 10592249 10.1093/nar/28.1.289 PMC102387

[33] Ashburner M, Ball CA, Blake JA, Botstein D, Butler H, et al (2000) Gene Ontology: tool for the unification of biology. Nature Genetics 25: 25–29. 10802651 10.1038/75556 PMC3037419

[34] Altman D (1991) Practical statistics for medical research. London, UK: Chapman & Hall/CRC.

